# Special Issue “Oral Health and Systemic Diseases”

**DOI:** 10.3390/jcm9103156

**Published:** 2020-09-29

**Authors:** Gerhard Schmalz, Dirk Ziebolz

**Affiliations:** Department of Cariology, Endodontology and Periodontology, University of Leipzig, 04103 Leipzig, Germany; gerhard.schmalz@medizin.uni-leipzig.de

With an enormous prevalence worldwide, diseases of the oral cavity and respective tissues are a highly relevant global health issue [1]. Beside of the oral health-related consequences of common oral diseases, like tooth loss, chewing deficiencies and quality of life impairment, oral and systemic disease interaction are apparent [2,3,4]. Different systemic diseases and conditions have the potential to affect a risk of developing oral diseases, especially periodontitis; on the other hand, oral inflammation can have an effect on general health. Moreover, several general diseases are related to a potential risk of complications in dental therapy [5].

The high variety and complexity of oral-systemic interactions is displayed by ten excellent articles in this special issue “Oral Health and Systemic Diseases”. Thereby, seven studies are reported, complemented by three systematic reviews. A case-control study by Gerreth et al. [6] comprehensively examined a group of 30 patients in the subacute phase of stroke regarding oral health and salivary parameters. For comparison, a matched control group was included. The authors presented an increase in protein glycooxidation, protein oxidation products, and lipid hydroperoxides in patients with stroke, indicating redox imbalances and oxidative damage to proteins and lipids in saliva of these patients. This leads to the recommendation of a close cooperation between dentists and medical professionals to provide comprehensive care in stroke patients. Furthermore, the diagnostic potential of saliva in stroke is highlighted. Machado et al. [7] included 1057 patients from the cohort of Study of Periodontal Health in Almada-Seixal (SoPHiAS), a Portuguese population-based cross-sectional study. The main objective of this investigation was the evaluation of associations between increased blood pressure and periodontitis. The study was able to confirm an association between high blood pressure and periodontitis within an adjusted logistic model. Moreover, the majority of patients with previously undetected increased blood pressure suffered from periodontal diseases. These results underline a close link between periodontitis and high blood pressure in this representative Portuguese population. In a study of seven clinical and immunhistochemical cases, Capodiferro et al. [8] examined metastases of renal cell carcinoma, which is the second most common metastatic carcinoma to the oro-facial tissues. These unusual neoplasms of the oral tissues require prompt and accurate diagnosis. Therefore, the clinical-pathological and immunological features of the reported seven clear cell renal cell carcinoma cases provide valuable information, which help to more accurately discriminate this neoplasm from other tumors of the oral cavity. The author group of Zieliński et al. [9] divided a cohort of 46 female participants into two groups (each *n* = 23) with or without depression based on the Research Diagnostic Criteria for Temporomandibular Disorders (RDC/TMDs) Axis II, respectively. All study participants underwent an electromyographical examination to assess their bioelectrical resting activity of temporal and masseter muscles. The resting activity of selected masticatory muscles did not differ between the two groups. The authors concluded that these preliminary findings should lead to further research with larger sample size to gain insight the psychological factors related to bioelectric parameters in masticatory muscles. A Polish study by Gerreth et al. [10] examined a cohort of 268 attendants of special-care schools with different degree of intellectual disability regarding enamel defects and their association to caries lesions. Nearly one fifth of the cohort was found to present developmental enamel defects, with the highest number of students in the subgroup showing mild intellectual disabilities. Active caries lesions, however, were more frequently detected in students without developmental defects. These contrary results need to be considered in further studies to understand the etiology of enamel defects in the dentition of individuals with a disability. A German cross-sectional study by Merle et al. [11] examined a group of 59 patients with juvenile idiopathic arthritis, a chronic rheumatic disease with an onset in childhood or youth. The majority of participants suffered from temporomandibular joint dysfunction and gingival inflammation. Moreover, high caries prevalence was reported. Additionally, c-reactive protein was related to gingival inflammation and radiographic findings in this cohort. Altogether, the authors conclude special dental care to be necessary for children and adolescents suffering from juvenile idiopathic arthritis. A prospective cohort study by Ziebolz et al. [12] was performed to assess whether a single recommendation of 88 patients with severe heart disease to visit their dentist leads to improved oral findings after 12 months follow-up. While the majority of participants followed the recommendation, only a slight reduction in periodontal treatment need was observed; thereby only 10% of individuals stated that they had received a periodontal therapy, albeit a periodontal treatment need was obvious in 91% of patients at baseline. Accordingly, a single recommendation to visit the dentist seems not to be adequate to ensure sufficient oral, especially periodontal conditions of patients with severe heart diseases. This also argues for the establishment of dental special care for these patients.

Three systematic reviews are part of this special issue. The systematic review by Pérez-Losada et al. [13] also applied a meta-analysis to detect, whether apical periodontitis would be associated to diabetes mellitus. Thereby, 21 studies, including ten studies in animals, ten studies dealing with human participants and one meta-analysis were identified. The analysis revealed an odds ratio for apical periodontitis and diabetes mellitus of 1.166 and 1.552 depending on the consideration of tooth- or patient-level, respectively. In conclusion, the association between diabetes mellitus and apical periodontitis was confirmed by this systematic review with meta-analysis. However, due to the medium or low quality of human studies, more prospective research in this field was concluded to be needed. Another systematic review was performed by the Spanish working group of Márquez-Arrico et al. [14] to reveal whether there is an association between polycystic ovary syndrome and chronic periodontitis. Ten articles were included after the review process and all of them confirmed significantly worse clinical periodontal parameters in patients with polycystic ovary syndrome. Accordingly, an association between periodontitis and polycystic ovary syndrome was confirmed by this systematic review. Moreover, the authors hypothesized a shared pathophysiology between the two diseases, based on a local and systemic proinflammatory environment and related oxidative stress. Finally, the third systematic review performed by Schmalz et al. [15] evaluated the oral health-related quality of life in adults with different systemic rheumatic diseases. Thereby, 26 studies with six different rheumatic entities were included. Generally, oral health-related quality was found to be reduced in patients with rheumatic diseases, whereby entities with oral primary manifestations, i.e., Sjögren Syndrome and Behcet disease most strongly affected the oral health-related quality of life. Additionally, patients with rheumatic diseases showed impairment in psychosocial impacts of oral health-related quality of life. Therefore, it was concluded that physical and psychosocial issues need to be addressed in patient centered dental care of these patients.

Altogether, the ten articles in the special issue “Oral Health and Systemic Diseases” show different aspects of oral and systemic disease interaction and provide several implications for dental care of these patients. One main conclusion is the necessity of further research, especially large-scaled prospective studies to get a better understanding of the needs of respective patient groups. Furthermore, patient centered dental special care appears recommendable for different patient groups, which were examined in the different studies. The special issue “Oral Health and Systemic Diseases” gives the reader an interesting and clinically relevant insight into oral and systemic disease interactions, an emerging field of dental and medical research.

## References

[1] Peres M.A., MacPherson L.M.D., Weyant R.J., Daly B., Venturelli R., Mathur M.R., Listl S., Celeste R.K., Guarnizo-Herreño C.C., Kearns C. (2019). Oral diseases: A global public health challenge. Lancet.

[2] Kumar P.S. (2016). From focal sepsis to periodontal medicine: A century of exploring the role of the oral microbiome in systemic disease. J. Physiol..

[3] Dörfer C., Benz C., Aida J., Campard G. (2017). The relationship of oral health with general health and NCDs: A brief review. Int. Dent. J..

[4] Winning L., Linden G. (2017). Periodontitis and Systemic Disease: Association or Causality?. Curr. Oral Health Rep..

[5] Schmalz G., Ziebolz D. (2020). Changing the Focus to the Whole Patient instead of One Oral Disease: The Concept of Individualized Prevention. Adv. Prev. Med..

[6] Gerreth P., Maciejczyk M., Zalewska A., Gerreth K., Hojan K. (2020). Comprehensive Evaluation of the Oral Health Status, Salivary Gland Function, and Oxidative Stress in the Saliva of Patients with Subacute Phase of Stroke: A Case-Control Study. J. Clin. Med..

[7] Machado V., Aguilera E.M., Botelho J., Hussain S.B., Leira Y., Proença L., D’Aiuto F., Mendes J.J. (2020). Association between Periodontitis and High Blood Pressure: Results from the Study of Periodontal Health in Almada-Seixal (SoPHiAS). J. Clin. Med..

[8] Capodiferro S., Limongelli L., Mastropasqua M.G., Favia G., Lajolo C., Colella G., Tempesta A., Maiorano E. (2020). Metastatic Tumors of the Oro-Facial Tissues: Clear Cell Renal Cell Carcinoma. A Clinico-Pathological and Immunohistochemical Study of Seven Cases. J. Clin. Med..

[9] Zieliński G., Byś A., Ginszt M., Baszczowski M., Szkutnik J., Majcher P., Gawda P. (2020). Depression and Resting Masticatory Muscle Activity. J. Clin. Med..

[10] Gerreth K., Opydo-Szymaczek J., Borysewicz-Lewicka M. (2020). A Study of Enamel Defects and Dental Caries of Permanent Dentition in School Children with Intellectual Disability. J. Clin. Med..

[11] Merle C.L., Hoffmann R., Schmickler J., Rühlmann M., Challakh N., Haak R., Schmalz G., Ziebolz D. (2020). Comprehensive Assessment of Orofacial Health and Disease Related Parameters in Adolescents with Juvenile Idiopathic Arthritis—A Cross-Sectional Study. J. Clin. Med..

[12] Ziebolz D., Friedrich S., Binner C., Rast J., Eisner M., Wagner J., Schmickler J., Kottmann T., Haak R., Borger M.A. (2020). Lack in Periodontal Care of Patients Suffering from Severe Heart Diseases—Results after 12 Months Follow-Up. J. Clin. Med..

[13] Pérez-Losada F.D.L., Estrugo-Devesa A., Castellanos-Cosano L., Segura-Egea J.J., López-López J., Velasco-Ortega E. (2020). Apical Periodontitis and Diabetes Mellitus Type 2: A Systematic Review and Meta-Analysis. J. Clin. Med..

[14] Márquez-Arrico C.F., Silvestre-Rangil J., Gutiérrez-Castillo L., Martínez-Herrera M., Silvestre F.J., Rocha M. (2020). Association between Periodontal Diseases and Polycystic Ovary Syndrome: A Systematic Review. J. Clin. Med..

[15] Schmalz G., Patschan S., Patschan D., Ziebolz D. (2020). Oral-Health-Related Quality of Life in Adult Patients with Rheumatic Diseases—A Systematic Review. J. Clin. Med..

